# The multidrug resistance 1 (*MDR1*) gene polymorphism G-rs3789243-A is not associated with disease susceptibility in Norwegian patients with colorectal adenoma and colorectal cancer; a case control study

**DOI:** 10.1186/1471-2350-10-18

**Published:** 2009-02-27

**Authors:** Vibeke Andersen, Lene Agerstjerne, Dorte Jensen, Mette Østergaard, Mona Sæbø, Julian Hamfjord, Elin Kure, Ulla Vogel

**Affiliations:** 1 Medical Department, Viborg Regional Hospital, 8800 Viborg, Denmark; 2 Department of Clinical Biochemistry, Viborg Regional Hospital, 8800 Viborg, Denmark; 3 Telemark University College, Faculty of Arts and Sciences, Department of Environmental and Health Studies, Hallvard Eikas plass, N-3800 Bø i Telemark, Norway; 4 Laboratory of Molecular Oncology, The Cancer Center, Oslo University Hospital, Ullevål, 0407 Oslo, Norway; 5 Section of Molecular Pathology, Department of Pathology, Oslo University Hospital, Ullevål, 0407 Oslo, Norway; 6 National Food Institute, Technical University of Denmark, DK-2860 Søborg, Denmark; 7 Institute for Science, Systems and Models, University of Roskilde, Denmark

## Abstract

**Background:**

Smoking, dietary factors, and alcohol consumption are known life style factors contributing to gastrointestinal carcinogenesis. Genetic variations in carcinogen handling may affect cancer risk. The multidrug resistance 1(*MDR1/ABCB1*) gene encodes the transport protein P-glycoprotein (a phase III xenobiotic transporter). P-glycoprotein is present in the intestinal mucosal lining and restricts absorption of certain carcinogens, among these polycyclic aromatic hydrocarbons. Moreover, P-glycoprotein transports various endogenous substrates such as cytokines and chemokines involved in inflammation, and may thereby affect the risk of malignity. Hence, genetic variations that modify the function of P-glycoprotein may be associated with the risk of colorectal cancer (CRC). We have previously found an association between the *MDR1 *intron 3 G-rs3789243-A polymorphism and the risk of CRC in a Danish study population. The aim of this study was to investigate if this *MDR1 *polymorphism was associated with risk of colorectal adenoma (CA) and CRC in the Norwegian population.

**Methods:**

Using a case-control design, the association between the *MDR1 *intron 3 G-rs3789243-A polymorphism and the risk of colorectal carcinomas and adenomas in the Norwegian population was assessed in 167 carcinomas, 990 adenomas, and 400 controls. Genotypes were determined by allelic discrimination. Odds ratio (OR) and 95 confidence interval (95% CI) were estimated by binary logistic regression.

**Results:**

No association was found between the *MDR1 *polymorphism (G-rs3789243-A) and colorectal adenomas or cancer. Carriers of the variant allele of MDR1 intron 3 had odds ratios (95% CI) of 0.97 (0.72–1.29) for developing adenomas, and 0.70 (0.41–1.21) for colorectal cancer, respectively, compared to homozygous wild type carriers.

**Conclusion:**

The *MDR1 *intron 3 (G-rs3789243-A) polymorphism was not associated with a risk of colorectal adenomas or carcinomas in the present Norwegian study group. Thus, this *MDR1 *polymorphism does not seem to play an important role in colorectal carcinogenesis in this population.

## Background

Colorectal cancer (CRC) is one of the leading causes of cancer-related mortality in the Western World, with great impact on the life quality of the affected persons [1]. Both genetic and environmental factors contribute to the pathogenesis, and gene-environmental interactions may modulate cancer risk. Multiple low-penetrance genes have been shown to confer susceptibility to CRC [2]. Diet components, alcohol consumption, and cigarette smoking are risk factors for CRC [3-6]. High-fat and low-fibre diets, red and processed meat consumption, and tobacco are all sources of carcinogenic heterocyclic amines (HCA) and polycyclic aromatic hydrocarbons (PAH) [7-9]. Alcohol consumption and cigarette smoking induce inflammation and may thereby contribute to carcinogenesis [10].

Genetic factors may influence the individual risk of gastrointestinal cancer when exposed to endogenous or exogenous carcinogens. The multidrug resistance 1 (*MDR1/ABCB1*) gene codes for P-glycoprotein, a membrane-bound transporter [11,12]. P-glycoprotein is abundant in the intestine [13,14] and transports a broad spectrum of substrates to the intestinal lumen [15], thereby constituting a part of the gastrointestinal barrier that protects the lining cells against xenobiotics, including possible carcinogens. One of the most potent animal PAH carcinogens, benzo [a]pyrene, may be a P-glycoprotein substrate [16]. Also various cytokines, such as interleukin-1beta, and chemokines involved in inflammation seem to be P-glycoprotein substrates [17], thus making a potential link between P-glycoprotein function and inflammation-induced carcinogenesis. *MDR1 *also seems to play a role in early carcinogenesis [18] by preventing apoptosis in tumor cells [17,19].

Significant *MDR1 *gene heterogeneity has been demonstrated [20,21]. The *MDR1 *C3435T polymorphism has been associated with low *in vivo *P-glycoprotein intestinal expression and activity in Caucasian subjects [22]. This is in accordance with the demonstration of an association of the variant allele with a lower mRNA stability [23]. This variant allele has been associated with higher risk of various clinical conditions [24], gastrointestinal cancers [25,26], and possibly colorectal cancer [27], although, generally, numbers of study participants in these studies have been small. However, results from other studies have been inconsistent [28-32], indicating that other causal polymorphism(s) may be implicated. Recently, we have demonstrated that carriers of the variant allele of *MDR1 *intron 3 (G-rs3789243-A) were at 1.54-fold higher risk of colorectal cancer than homozygous wild type carriers (95% CI: 1.13–2.08) in a case-cohort study nested in the prospective Danish Diet, Cancer and Health cohort study (V. Andersen et al, submitted). This variant has previously been found to be associated with the risk of ulcerative colitis by the use of a haplotype tagging approach [33]. So far, the SNP has no known functional effects [33].

We wished to explore the role of the xenobiotic transporter P-glycoprotein, encoded by the *MDR1 *gene and known to transport dietary carcinogens, in CRC etiology. Nearly all colon cancers begin as benign polyps which slowly develop into cancer. We speculated that the uptake of dietary carcinogens via P-glycoprotein may be involved in this process. Therefore, the role of genetic variation in *MDR1 *intron 3 (G-rs3789243-A) in relation to the risk of developing colorectal adenomas and carcinomas was assessed in the Norwegian population using a case-control study of 167 carcinomas, 990 adenomas, and 400 controls.

## Methods

The KAM (Kolorektal cancer, Arv og Miljø) cohort is based on the screening group of the Norwegian Colorectal Cancer Prevention study (the NORCCAP study) in the county of Telemark and a series of clinical CRC cases operated at Telemark Hospital (Skien) and Ulleval University Hospital (Oslo) [34,35]. In short, 20.780 healthy men and women, age 50–64 years of age, drawn at random from the population registry in Oslo (urban) and the county of Telemark (mixed urban and rural) were invited to have a flexible sigmoidoscopy screening examination. The KAM cohort is based on an ethnically homogeneous group of Norwegian origin.

The KAM biobank consists of samples from individuals identified with adenomas in the large intestine (1044 accepted; 991 high- and low-risk adenomas (a high-risk adenoma being defined as an adenoma measuring at least 10 mm in diameter and/or with villous components and/or showing severe dysplasia), and 53 hyperplastic polyps), and controls, defined as individuals with normal findings at flexible sigmoidoscopy screening (400 accepted), together with 167 cases identified with colorectal cancer. All of the participants completed a questionnaire on demographics, health status, dietary and smoking habits, alcohol consumption, physical exercise and occupation. The questionnaire contained information on a family history of adenomas and carcinomas, and the included cases and controls had no known personal history of genetic predisposition. In the present study, blood samples were available from 167 cases with carcinomas, 990 cases with adenomas (229 high-risk and 761 low-risk adenomas) and 400 controls. All participants gave verbal and written informed consent.

The study was done in accordance with the Helsinki Declaration. The Regional Ethics Committee and the Data Inspectorate approved the KAM study. The ID number for the study is NCT00119912 at ClinicalTrials.gov [36].

The distribution of age, gender, smoking, and alcohol consumption among cases and controls are shown in Table 1.

**Table 1:** Characteristics of study participants with colorectal carcinomas and high- and low-risk adenomas and healthy controls, in total 1557 subjects.

	**Colorectal carcinomas**	**High-risk adenomas**	**Low-risk adenomas**	**Controls**
**No. of subjects**	167	229	761	400
**Gender**				
Male, No (%)	91 (56)	151 (66)	455 (60)	157 (39)
Female, No (%)	76 (44)	78 (34)	306 (40)	243 (61)
				
				
**Age at inclusion, median**	69 (51–86)	57 (53–63)	57 (51–63)	53 (50–63)
				
**BMI, median**	25 (19–31)	26 (21–33)	27 (21–32)	26 (21–32)
				
**Red meat, g/day, median**	24 (5–76)	27 (7–86)	27 (5–90)	25 (5–95)
				
**Smoking status**				
Never, No (%)	39 (33)	52 (27)	206 (32)	156 (47)
Ever, No (%)	80 (67)	140 (73)	448 (68)	178 (53)

Observed median values (5–95 percentiles) or percents of potential colorectal cancer confounders among cases and controls.

Genomic DNA was isolated from blood samples according to standard procedures [37] with minor modifications as already described [38]. All analyses were run blinded to the case-control status. *MDR1 *G-rs3789243-A [GenBank:rs3789243]genotyping was performed on an Mx3000 machine (Stratagene, La Jolla, CA, USA), using allelic discrimination with TaqMan chemistry, as earlier described [39]. In short, QPCR was done in 25 μl reactions including 20 ng DNA and 900 nM primers and 200 nM probes with locked nucleic acid (LNA) incorporations. QPCR was run for 50 cycles with split annealing and elongation (62°C and 72°C, respectively). Controls were included in each run. Within each of the three genotype groups, 20 randomly selected samples were repeated to confirm reproducibility.

The genotype frequencies were compared using a logistic regression model, measured as odds ratio (OR) with 95% confidence interval (CI). Two separate ORs were calculated; one crude (OR^a) and one adjusted for age and gender (OR^b). In addition we analyzed for carriers of the polymorphism separately. MiniTab Statistical Software, Release 13.1 Xtra (Minitab Inc.) for Windows was used for the statistical calculations Given the allele frequency of 0.5, we had 80% chance of detecting an OR of approximately 1.7 in carcinomas and 1.5 in adenomas at a 5% significance level [40].

## Results

Characteristics of the study population and risk factors for CRC are shown in Table 1. Women were more frequent among the controls than among cases. Smoking was more frequent among cases than among controls. Moreover, the CRC cases were older than the controls. The genotype distribution among the controls did not deviate from Hardy-Weinberg equilibrium. No association was found between the *MDR1 *polymorphism (G-rs3789243-A) and the risk of colorectal adenomas or cancer (Table 2). Carriers of the variant allele in *MDR1 *intron 3 had ORs (95% CI) of 0.96 (0.72–1.29) for developing adenomas, and 0.70 (0.41–1.21) for developing colorectal cancer, respectively, compared to homozygous wild type carriers.

**Table 2:** Odds Ratios for Colorectal Cancers and Colorectal Adenomas including high- and low-risk adenomas for *MDR1 *G-rs3789243-A

	**N_Case_**	**N_Control_**	**OR^a^**	**95% CI**	**OR^b^**	**95% CI**
**Colorectal Cancer**						
GG	50	101	1.00	-	1.00	-
GA	78	190	0.80	(0.53–1.22)	0.79	(0.44–1.41)
AA	39	104	0.73	(0.45–1.20)	0.55	(0.27–1.12)
GA and AA	117	294	0.78	(052–1.15)	0.70	(0.41–1.21)
**Colorectal Adenomas**						
GG	253	101	1.00	-	1.00	-
GA	482	190	1.01	(0.76–1.35)	0.98	(0.73–1.35)
AA	234	104	0.90	(0.65–1.24)	0.93	(0.65–1.33)
GA and AA	716	294	0.97	(0.74–1.27)	0.96	(0.72–1.29)
**High-risk adenomas**						
GG	61	101	1.00	-	1.00	-
GA	109	190	0.95	(0.64–1.41)	1.08	(0.69–1.70)
AA	54	104	0.86	(0.54–1.36)	0.98	(0.58–1.64)
GA and AA	163	294	0.92	(0.63–1.33)	1.04	(0.68–1.59)
**Low-risk adenomas**						
GG	192	101	1.00	-	1.00	-
GA	373	190	1.03	(0.77–1.39)	0.98	(0.71–1.36)
AA	180	104	0.91	(0.65–1.28)	0.93	(0.64–1.36)
GA and AA	553	294	0.99	(0.75–1.31)	0.97	(0.71–1.32)

^a^Crude.

^b^Adjusted for age and gender.

## Discussion

We found no associations between the studied *MDR1 *polymorphism and the risk of colorectal adenomas or carcinomas in the present Norwegian study group. The risk of colorectal adenomas and carcinomas were not different for homozygous or heterozygous carriers of the variant genotype of *MDR1 *G-rs3789243-A compared with the homozygous wild type carriers. A power calculation showed that we had 80% chance of detecting an OR of approximately 1.7 in carcinomas and 1.5 in adenomas. The statistical power to look into interactions was therefore limited, and interaction studies were therefore not done.

*MDR1 *genotypes have previously been studied in relation to colorectal cancer susceptibility [27,41,42]. In contrast to the present study, variant allele carriers of the *MDR1 *G-rs3789243-A polymorphism have previously been associated with a risk of colorectal cancer in the Danish population (V. Andersen et al, submitted) and of ulcerative colitis in the Scottish population [33]. In the Danish study, carriers of the variant allele were at 1.54-fold higher risk of CRC compared with wild type carriers (95% CI: 1.13–2.08).

The *MDR1 *C3435T polymorphism was studied in a Polish study population [27]. They found higher risk of CRC among variant carriers than homozygous wildtype carriers in a subgroup of younger persons. However, as the result was based on a subgroup consisting of about 50 persons the findings need to be replicated in a larger study. Furthermore, no association between this polymorphism and colon cancer was reported in a Korean study population [41].

Several factors may contribute to the differences seen between the studies. First of all, the first report of genetic effects is likely to represent an overestimated odds ratios due to reporting bias [43]. Therefore, the association may be real but not reproduced due to a weak gene effect and limited power to detect such small gene effects in the Norwegian replication study.

Population heterogeneity may also contribute. However, although *MDR1 *shows a high degree of heterogeneity between different population [20,21,44], it may be suggested that the Norwegian, Scottish and Danish population may share some common genetic background as it seems to be the case for *CARD15 *polymorphisms in European populations [45-47], where a low frequency of the *CARD15 *variants in the Northern countries compared to the rest of Europe has been demonstrated.

Furthermore, differences in carcinogenic exposure may modify the inherent risk associated with genetic susceptibility. For instance, the average monthly intake of alcohol was 11 units, corresponding to 88 g alcohol per month among the controls in the present Norwegian [38] cohort, whereas the mean alcohol intake was 390 g per month among the controls in the Danish cohort [48]. Correspondingly, 47% and 34% of the controls were never smokers in the Norwegian and Danish studies, respectively. However, due to the restricted sample size such differences were not elaborated.

## Conclusion

The *MDR1 *G-rs3789243-A polymorphism was not associated with risk of colorectal adenomas or carcinomas in the present Norwegian study group, indicating that this polymorphism does not play an important role in the colorectal carcinogenesis in the Norwegian population.

## Abbreviations

CA: Colorectal Adenoma; CRC: Colorectal Cancer; HCA: heterocyclic amines; *MDR1*: Multidrug Resistance 1; OR: odds ratio; CI: confidence interval; PAH: polycyclic aromatic hydrocarbons; QPCR: real-time quantitative PCR; SNP: single nucleotide polymorphism; IRR: incidence rate ratio.

## Competing interests

The authors declare that they have no competing interests.

## Authors' contributions

LA, DJ, MØ carried out the genotyping. VA and LA drafted the manuscript. EK established the KAM study. EK, MS and JH participated in sample preparation and collection, MS performed the statistical analyses and JH administered the KAM database. VA and UV conceived the genotyping study, and participated in its design and coordination and helped to draft the manuscript. All authors read and approved the final manuscript.

## Pre-publication history

The pre-publication history for this paper can be accessed here:


